# Ryanodine Receptors in Autophagy: Implications for Neurodegenerative Diseases?

**DOI:** 10.3389/fncel.2018.00089

**Published:** 2018-03-27

**Authors:** Tim Vervliet

**Affiliations:** Laboratory of Molecular and Cellular Signaling, Department of Cellular and Molecular Medicine, KU Leuven, Leuven, Belgium

**Keywords:** ryanodine receptor, autophagy, Ca^2+^ signaling, neurodegenerative diseases

## Abstract

Intracellular Ca^2+^ signaling is important in the regulation of several cellular processes including autophagy. The endoplasmic reticulum (ER) is the main and largest intracellular Ca^2+^ store. At the ER two protein families of Ca^2+^ release channels, inositol 1,4,5-trisphosphate receptors (IP_3_Rs) and ryanodine receptors (RyRs), are expressed. Several studies have reported roles in the regulation of autophagy for the ubiquitously expressed IP_3_R. For instance, IP_3_R-mediated Ca^2+^ release supresses basal autophagic flux by promoting mitochondrial metabolism, while also promoting the rapid initial increase in autophagic flux in response to nutrient starvation. Insights into the contribution of RyRs in autophagy have been lagging significantly compared to the advances made for IP_3_Rs. This is rather surprising considering that RyRs are predominantly expressed in long-lived cells with specialized metabolic needs, such as neurons and muscle cells, in which autophagy plays important roles. In this review article, recent studies revealing roles for RyRs in the regulation of autophagy will be discussed. Several RyR-interacting proteins that have been established to modulate both RyR function and autophagy will also be highlighted. Finally, the involvement of RyRs in neurodegenerative diseases will be addressed. Inhibition of RyR channels has not only been shown to be beneficial for treating several of these diseases but also regulates autophagy.

## Introduction

Intracellular Ca^2+^ signaling regulates a wide array of cellular processes such as cell division, muscle contraction, memory formation, secretion, cell death, autophagy… (Berridge, 2001). Although many different organelles are involved in Ca^2+^ signaling, the endoplasmic reticulum (ER) functions as the main intracellular Ca^2+^ store. Within the lumen of the ER maintaining adequate Ca^2+^ levels is essential to promote the correct folding of proteins. Failure to do so results in ER stress and activation of the unfolded protein response (Mekahli et al., 2011; Carreras-Sureda et al., 2018). In response to extracellular stimuli and agonists such as growth factors, hormones, neurotransmitters and antibodies, Ca^2+^ release from the ER is a common extensively regulated event (Berridge, 2001). Ca^2+^ responses can occur in highly localized microdomains, enabling intimate Ca^2+^ signaling between different organelles like the ER and mitochondria and the ER and lysosomes (Laude and Simpson, 2009; Kilpatrick et al., 2017; Pedriali et al., 2017) or they can spread across the entire cell as Ca^2+^ oscillations and waves (Thul et al., 2008). Depending on the spatio-temporal profile of the Ca^2+^ response, different processes are affected (Berridge et al., 2003). Long lasting Ca^2+^ oscillations, for instance are known to promote cell survival by stimulating mitochondrial ATP production, whereas excessive Ca^2+^ release is associated with cell death.

The cellular components involved in ER Ca^2+^ dynamics have been studied extensively and are well-characterized in terms of function and regulation. Ca^2+^ is actively transported into the ER via the sarco/endoplasmic reticulum Ca^2+^ ATPase (SERCA; Vandecaetsbeek et al., 2011). The majority of Ca^2+^ release from the ER is mediated by two families of Ca^2+^-permeable channels, inositol 1,4,5-trisphosphate (IP_3_) receptors (IP_3_Rs; Foskett et al., 2007; Parys and De Smedt, 2012; Ivanova et al., 2014; Mikoshiba, 2015) and ryanodine receptors (RyRs; discussed in more detail later in this review article). IP_3_R are a class of tetrameric intracellular Ca^2+^-release channels gated by the second messenger IP_3_ (Parys and De Smedt, 2012). IP_3_R-mediated Ca^2+^ signaling is present in virtually all cells and is involved in the regulation of mitochondrial bioenergetics, autophagy, cell death… (Cárdenas et al., 2010, 2016; Bultynck, 2016). Besides IP_3_Rs and RyRs, other proteins involved in ER Ca^2+^ release or leak pathways are Bax Inhibitor-1, pannexin-1, translocon, presenilins and Orai2 (Sammels et al., 2010b). Apart from the ER, the mitochondria, lysosomes and the golgi apparatus are known to sequester Ca^2+^ and contribute to Ca^2+^ signaling events (Raffaello et al., 2016). ER, mitochondria and lysosomes are also important organelles in the regulation of autophagy (La Rovere et al., 2016). It is therefore not surprising that Ca^2+^ signaling is involved in the regulation of autophagy (Decuypere et al., 2011a).

## Ca^2+^ Signaling in Autophagy

Autophagy is a degradation pathway responsible for maintaining proper cell homeostasis. It removes damaged organelles or helps cells cope with stress situations such as starvation, the accumulation of unfolded proteins or infections. Three forms of autophagy, micro-, macro- and chaperone-mediated autophagy, are known to exist which all end with degradation in the lysosomes (Decuypere et al., 2011a). Macroautophagy (from here on referred to as autophagy) is a complex signaling pathway involving several steps which guide the formation, elongation, maturation and finally fusion of phagophores with lysosomes. Several of these steps are Ca^2+^ dependent (Decuypere et al., 2011a). Both stimulatory and inhibitory effects of Ca^2+^ on autophagy have been described, which likely reflects a strict spatio-temporal control of autophagy by Ca^2+^ signaling, recently discussed in great detail in Bootman et al. (2018). In Brief, Ca^2+^ influx through voltage-gated Ca^2+^ channels has been shown to inhibit autophagy by activating calpains that cleave ATG5, which is important for autophagosome elongation (Williams et al., 2008; Xia et al., 2010). As for stimulatory effects, several studies have shown that increased cytosolic Ca^2+^ levels trigger the activation of calmodulin, thus activating the AMP-activated protein kinase (AMPK) via calmodulin kinase kinase β leading to the inhibition of the mechanistic target of rapamycin (mTOR), thereby inducing autophagy (Høyer-Hansen et al., 2007; Law et al., 2010).

At the level of the ER, the IP_3_R was shown to be involved in the regulation of autophagy. A continuous Ca^2+^ transfer from the ER to the mitochondria is necessary to maintain proper ATP production thereby inhibiting the activation of AMPK and thus inhibiting autophagy (Cárdenas et al., 2010). In particular, cancer cells appear to depend critically on these constitutive ER-mitochondrial Ca^2+^ fluxes for their survival, since genetic or pharmacological interference with such fluxes results in cancer cell death (Bultynck, 2016; Cárdenas et al., 2016). For a more in-depth review on the regulation of autophagy by IP_3_Rs, I would like to refer to the following reviews (Parys et al., 2012; Kania et al., 2017).

## The Ryanodine Receptor (RyR)

RyRs are large (>500 kDa) ER-located Ca^2+^-permeable channels of which three isoforms are known to exist. These channels form and function as tetramers (Lanner et al., 2010). In contrast to the ubiquitously expressed IP_3_Rs, RyRs have a distinct expression pattern restricted to specific cell types such as T-cells and excitable cells. Tissues expressing RyRs include heart, skeletal and smooth muscle, brain, pancreas and the liver. From these tissues, skeletal muscles, heart and neurons have the highest RyR-expression levels (Lanner et al., 2010). More specifically, skeletal muscle cells express large amounts of RyR1 (Lai and Meissner, 1989), heart cells express RyR2 (Imagawa et al., 1989) whereas all three RyR isoforms are expressed in different areas of the brain (Martin et al., 1998). Given this rather restricted expression pattern, it is not surprising that RyRs are involved in several tissue-specific processes. In muscle and heart cells, RyR1 and RyR2 are critically involved in excitation-contraction coupling. In neurons long term potentiation and long term depression are known to be at least in part RyR dependent (Lanner et al., 2010). In the pancreas, RyRs are involved in the secretion of insulin (Santulli et al., 2015) and they also contribute to the development of bile acid-induced acute pancreatitis (Husain et al., 2012). Physiological RyR activators are Ca^2+^, cyclic-ADP ribose and NAADP (Meissner et al., 1997; Kunerth et al., 2004; Gerasimenko et al., 2006). However, it remains unclear whether NAADP activates RyRs directly or via Ca^2+^-induced Ca^2+^ release (CICR) initiated at the level of the lysosomes via NAADP-sensitive Ca^2+^ channels. Pharmacologically RyRs can be activated by caffeine or low ryanodine concentrations (nM range), locking the channel in a sub-conductance state. Inhibition of RyRs can be obtained most specifically via a high ryanodine conentrations (>20 μM) or with dantrolene, a FDA-approved RyR blocker.

## Regulation of the RyR by Protein-Protein Interactions

RyR channels are regulated by many different cellular components (Ca^2+^, Mg^2+^ and ATP), post translational modifications and protein interactions (Lanner et al., 2010). In this section, we will focus on a number of proteins that regulate RyR-mediated Ca^2+^ release, which may potentially impact its role in autophagy.

### B-Cell Lymphoma (Bcl)-2 and Bcl-X_L_

The Bcl-2-protein family is well known as critical regulator of apoptosis. This family consists of anti- and pro-apoptotic members, which are all characterized by the presence of at least one and maximum four Bcl-2 homology (BH) domains (Letai, 2008). Anti-apoptotic Bcl-2 and Bcl-X_L_ bind to pro-apoptotic family members thereby preventing apoptosis-induction at the mitochondria (Brunelle and Letai, 2009). In addition to the regulation of apoptosis at the level of the mitochondria, Bcl-2 proteins have emerged as critical modulators of intracellular Ca^2+^-transport systems and Ca^2+^ signaling events (Vervliet et al., 2016). Both Bcl-2 and Bcl-X_L_ have been extensively shown to regulate IP_3_R-mediated Ca^2+^ release in several manners (Chen et al., 2004; White et al., 2005; Hanson et al., 2008; Rong et al., 2009; Eckenrode et al., 2010; Monaco et al., 2012; Yang et al., 2016). Other Ca^2+^-signaling related targets of anti-apoptotic Bcl-2 proteins include the plasma membrane Ca^2+^ ATPase (Ferdek et al., 2012), SERCA (Dremina et al., 2004) and the voltage dependent anion channel (VDAC; Shimizu et al., 1999).

Recently, we have shown that both Bcl-2 and Bcl-X_L_ bind to and inhibit RyRs (Vervliet et al., 2014, 2015a). Both proteins bound to a centrally located region on the RyR, showing a striking similarity to the Bcl-2 binding site in the central domain of the IP_3_R (Rong et al., 2009). The fourth BH (BH4) domain of Bcl-2 and Bcl-X_L_ was sufficient for inhibiting RyR-mediated Ca^2+^ release. However, in full-size Bcl-X_L_, also the BH3 domain and more specifically lysine 87, within this domain, was important for targeting and inhibiting RyR activity.

### Presenilins

Presenilins form the catalytic subunit of γ-proteases. Substrates of presenilins include notch and the amyloid precursor protein (De Strooper et al., 2012). Presenilins are extensively studied for their role in the development of Alzheimer’s disease (AD; Selkoe and Hardy, 2016). In AD, presenilin mutations alter the cleavage of the amyloid precursor protein, enhancing the formation of pathological amyloid beta. This pathological amyloid beta aggregates and forms plaques that are considered as an important hallmark of the disease. Besides their actions as proteases, presenilins are also known to play roles in Ca^2+^ signaling. At the ER, presenilins have been implicated in inducing a passive ER Ca^2+^ leak. It was proposed that these proteins can form Ca^2+^-permeable pores potentially functioning as a Ca^2+^-leak channel (Nelson et al., 2007). This unconventional role for presenilins as Ca^2+^-leak channels also surfaced in an unbiased systems biology approach using a collection of siRNAs against Ca^2+^ regulators in living single cells (Bandara et al., 2013). Presenilins also regulate IP_3_R channel function and expression, which may contribute to their ER Ca^2+^ leak properties (Kasri et al., 2006; Cheung et al., 2010; Wu et al., 2013).

RyR expression and function is also dependent on presenilins. Loss of presenilins in primary hippocampal neurons resulted in a decrease in RyR expression and reduced RyR-mediated Ca^2+^ release, impairing pre-synaptic function (Wu et al., 2013). In addition, an N-terminal fragment of presenilin-1 was shown to inhibit RyR-mediated Ca^2+^ release, thereby directly regulating RyR channel activity (Payne et al., 2013).

### Polycystins (PC)

Polycystin (PC) 1 and 2 are important regulators of intracellular Ca^2+^ signaling (Lemos and Ehrlich, 2018). Both PC1 and PC2 are present at ER membranes, plasma membranes and cilia. PC2 is a non-selective Ca^2+^-permeable cation channel, whereas PC1 is important for PC2 localization and functioning (Tsiokas et al., 2007; Delling et al., 2013). Loss of function of either PC results in the development of polycytic kidney disease, a disease characterized by aberrant intracellular Ca^2+^ signaling leading to cyst growth and ultimately kidney failure (Torres et al., 2007). These alterations in Ca^2+^ homeostasis are generally attributed to loss of correct PC2 functioning or altered regulation of different Ca^2+^ release channels by PC1 or PC2. The IP_3_R for instance is regulated by both PC1 and PC2. PC1 interacts with the IP_3_R thereby inhibiting IP_3_R-mediated Ca^2+^ release whereas PC2 mediates the opposite effect on the IP_3_R (Li et al., 2005, 2009; Sammels et al., 2010a).

In cardiomyocytes, RyR2 is regulated by PC2 (Anyatonwu et al., 2007). It was shown that PC2 interacts with RyR2, thereby inhibiting the channel. Knock out of PC2 resulted in increased frequency of Ca^2+^ oscillations in the cardiomyocytes and reduced the ER Ca^2+^ store content. Recently, it was shown that a decrease in PC2 expression increased the expression of RyR2 and SERCA2A in the heart while phosphorylated phospholamban, a SERCA inhibitor in its non-phosphorylated form, was decreased (Kuo et al., 2014). These results indicate that PC2 regulates RyRs and Ca^2+^ signaling in the heart at multiple levels.

## The RyR in Autophagy

As mentioned above intracellular Ca^2+^ signaling is a versatile regulator of autophagy. Major advances on the regulation of autophagic flux by IP_3_Rs in nutrient-rich and starvation conditions but also in response to treatments with for instance rapamycin and resveratrol have been made (Kania et al., 2017). However, our understanding of how RyR-mediated Ca^2+^ release modulates the autophagic process has lagged compared to the progress made for IP_3_Rs. A major issue for studying the role of RyRs in autophagy has been the lack of easy-to-use cell models that express RyRs endogenously. As autophagy is easily influenced by many factors, expressing exogenous RyRs in cells that do not have adequate RyR regulators, may alter autophagy and other processes in a way that may have little physiological relevance. Therefore, it is important to include model systems expressing endogenous RyRs as well, to assess whether or not the observations are replicable in a more physiological setting. That being said, high RyR expression is observed in skeletal muscle, heart and brain cells, which are all long-lived cells with specific metabolic needs where autophagy plays important roles. In addition, in these tissues RyR-mediated Ca^2+^ release is important for performing several main functions like muscle contraction and neuronal processes related to synaptic transmission and memory formation. As RyRs are activated by CICR (Lanner et al., 2010) they may serve to amplify Ca^2+^ signals or initiate local Ca^2+^ signals that contribute to the sensitization of Ca^2+^-handling systems promoting ER Ca^2+^ release thus modulating autophagy in a positive or negative way (Bootman et al., 2018). Recently, a number studies have emerged, thereby revealing a role for RyR channels in autophagy regulation.

First, the involvement of RyR3 in autophagic cell death, a non-apoptotic form of programmed cell death driven by autophagy (Edinger and Thompson, 2004), was demonstrated in adult neuronal hippocampal stem cells (Chung et al., 2016). The authors showed that in these cells, insulin withdrawal increased the protein levels of LC3-II, an important autophagic flux marker (Klionsky et al., 2016), suggesting a stimulation of the autophagic flux. Insulin deprivation also upregulated RyR1 and RyR3-mRNA correlating with increased Ca^2+^ release from the ER, resulting in elevated cytosolic Ca^2+^ levels. Pharmacological inhibition of RyRs using dantrolene could counteract the increase in LC3-II levels. It is likely that RyR3 upregulation was a toxic event that contributed to the observed autophagic cell death induced by insulin withdrawal, as dantrolene could protect against this, while caffeine had the opposite effect. These findings were supported by CRISPR/Cas9-mediated knockout of RyR3 in these neuronal stem cells, indicating that the RyR3-deficient stem cells did not display the observed Ca^2+^ rises and were protected from autophagic cell death in response to insulin withdrawal.

The effects of propofol, a commonly used intravenous anesthetic known to induce neuronal damage and learning deficits in rat (Milanovic et al., 2010; Karen et al., 2013), on autophagy regulation have also been reported recently (Qiao et al., 2017). Treatment of cortical progenitor cells with a high dose of propofol resulted in cytotoxicity which was dependent on both IP_3_R and RyR activation. The authors linked this cytotoxicity to excessive autophagy activation, measured by increased LC3-II levels after propofol treatment. Inhibition of either IP_3_Rs or RyRs, using xestospongin C or dantrolene respectively, reduced LC3-II to control levels and increased cell viability. Suggesting RyRs are involved in the excessive induction of autophagy by propofol.

In further recent work, we showed in HEK RyR3-overexpression models, differentiated C2C12, cells and dissociated hippocampal neurons that spontaneous RyR activity inhibits basal autophagic flux (Vervliet et al., 2017). By blocking RyR activity with either dantrolene or an inhibitory dose of ryanodine, a reduction in LC3-II levels was observed. Including bafilomycin A1, a lysosomal degradation blocker, under these conditions increased LC3-II to similar levels as the control conditions. This suggested that the observed decrease in LC3-II levels upon RyR inhibition was due to an increased turnover of autophagosomes. Early markers of autophagy induction such as Beclin1, mTOR and AMPK activity were unaltered, confirming that inhibiting sporadic RyR-mediated Ca^2+^ release increases autophagic flux at the level of the lysosomes.

Although all three above studies reported a reduction in LC3-II levels upon RyR inhibition seemingly contradicting conclusions were drawn concerning the effects of RyR inhibition on autophagic flux. In the first two studies a decrease in autophagic flux was shown (Chung et al., 2016; Qiao et al., 2017), whereas the last study suggests a stimulation of autophagic flux upon RyR inhibition (Vervliet et al., 2017). A major difference between these studies is whether or not autophagy stimulating treatments or agents were used. The first two studies thus addressed the role of RyRs in autophagy induction by an external trigger, insulin deprivation or propofol treatment (Chung et al., 2016; Qiao et al., 2017). The third study focused on basal autophagy and spontaneous RyR activity (Vervliet et al., 2017). Although the conclusions of these studies appear seemingly contradictory, it is important to note that the role of Ca^2+^ signaling in basal vs. induced autophagy can be completely different and even opposite which has been illustrated for IP_3_Rs (Cárdenas et al., 2010; Decuypere et al., 2011b; Bootman et al., 2018).

Another recent study showed that RyR inhibition using dantrolene attenuated the observed alterations in autophagy in neurons from a mouse model for Gaucher disease (Liou et al., 2016). In this mouse model, neuronal RyR3 levels were reduced. In addition, an increase in LC3-II levels was observed compared to control mice. Treating these mice with dantrolene reduced the LC3-II levels compared to the untreated controls, improved mitochondrial function and overall survival rates. Strikingly, dantrolene treatment resulted in an increase in RyR3 levels to near WT levels. The authors concluded that dantrolene treatment could largely reverse the observed defects in autophagy in these mice, thereby normalizing basal autophagy.

In the heart RyR activity has been shown to regulate mitochondrial metabolism. Downregulation of RyR2 in cardiomyocytes resulted in decreased mitochondrial metabolism decreasing ATP production (Bround et al., 2013). Reduction of RyR2 triggered a hypoxia-like state thereby upregulating hypoxia inducible factor (Hif1α), and presenilin-1 and -2, and also increased autophagy induction. Reduced RyR2 levels lead to a calpain 10-dependent form of non-apoptotic programmed cell death, which has also been described to occur in pancreatic β-cells treated with RyR inhibitors (Dror et al., 2008). This study links RyR2-mediated Ca^2+^ release directly to mitochondrial metabolism, ATP production and the induction of autophagy. It is important to note that during ischemia reperfusion RyR2 levels in the heart drop dramatically due to calpain activity, proteasomal degradation and chaperone-mediated autophagy (Pedrozo et al., 2013). This was proposed to be protective as mitochondrial metabolism is slowed down, resulting in less production of reactive oxygen species. This study also showed that reducing RyR2 levels in cardiomyocytes triggers autophagy and may thus confer additional protection for cardiomyocytes. However, reduced RyR2 levels may impair cardiac contractility, which could lead to heart failure.

## RyR Regulators in Autophagy

### Bcl-2/Bcl-X_L_

Anti-apoptotic Bcl-2 proteins are known to inhibit autophagy by sequestering Beclin 1 thereby inhibiting its pro-autophagic actions (Pattingre et al., 2005). This interaction occurs via the hydrophobic cleft of the Bcl-2 proteins and a BH3-like domain in Beclin 1 (Pedro et al., 2015). As Bcl-2 binds to Beclin 1 via its hydrophobic cleft and to the IP_3_R via its BH4 domain, it is possible that Bcl-2 sequesters Beclin 1 at the IP_3_R (Decuypere et al., 2011a). When Beclin 1 is then released from Bcl-2 it would be in close proximity of the IP_3_R, where it may sensitize IP_3_R-mediated Ca^2+^ release thereby promoting starvation-induced autophagy. At this point it is not known whether Bcl-2 bound to RyRs is associated with Beclin 1 and thus whether Beclin 1 can be found in a complex with RyRs. This is possible since Bcl-2 binding to the RyRs is independent of its hydrophobic cleft (Vervliet et al., 2015b). The hydrophobic cleft of Bcl-2 proteins associated with RyRs might thus be available for other BH3-domain-containing proteins, like Beclin 1. It will be of interest to investigate whether Beclin 1 can bind to RyR channels directly or indirectly (e.g., via Bcl-2) and whether such complex formation impacts the function of the RyR in autophagy.

Bcl-2 and Bcl-X_L_ both regulate IP_3_R-mediated Ca^2+^ release (White et al., 2005; Rong et al., 2009; Decuypere et al., 2011a; Monaco et al., 2012) and mitochondrial Ca^2+^ uptake via VDAC (Shimizu et al., 1999, 2000; Arbel and Shoshan-Barmatz, 2010). These proteins have a great impact on mitochondrial bioenergetics and ATP production and may also impact autophagy in this manner. As RyR-mediated Ca^2+^ release also regulates mitochondrial metabolism (Bround et al., 2013), inhibition of RyRs via Bcl-2 and Bcl-X_L_ potentially also modulates autophagy through the regulation of RyR-mediated Ca^2+^ transfer to the mitochondria in certain cells. In addition, our data suggest that spontaneous RyR-mediated Ca^2+^ release inhibits the autophagic flux at the level of the lysosomes (Vervliet et al., 2017). As such, Bcl-2 and Bcl-X_L_ could potentially promote autophagy by inhibiting basal RyR function.

### Presenilins

In the heart, it was shown that downregulation of RyR2 induced a hypoxic-like condition associated with upregulation of presenilins (Bround et al., 2013). Similar observations were made in pancreatic β-cells where RyR inhibition resulted in depletion of ATP and a presenilin-dependent induction of Hif1α (Dror et al., 2008). This suggests a role for presenilins in regulating mitochondrial metabolism under stress conditions.

AD-associated presenilin mutants have been shown to increase IP_3_R-mediated Ca^2+^ release (Cheung et al., 2010). This may have direct effects on mitochondrial metabolism, energy production and autophagy (Bootman et al., 2018). RyR expression levels and functions are also regulated by presenilins (Payne et al., 2013; Wu et al., 2013). Knock down of presenilins for instance, reduced RyR expression levels in hippocampal neurons which in turn lowered RyR-mediated Ca^2+^ release in these neurons (Wu et al., 2013). This decrease in RyR signaling could potentially also regulate autophagic flux. However, at the moment it is not known whether presenilins regulate autophagy by targeting ER Ca^2+^ release channels.

### Polycystins

PC1 and PC2 influence autophagy in a number of ways. First, they both control the activity of mTOR, an important kinase critically involved in autophagy induction (Laplante and Sabatini, 2012). Overexpression of PC1 resulted in decreased mTOR activity (Distefano et al., 2009) whereas either PC1 or PC2 knock-down increased mTOR activity (Rowe et al., 2013; Ravichandran et al., 2015; Orhon et al., 2016). In polycystic kidney disease, mTOR signaling is increased which correlates well with loss of function of PC1 and PC2 proteins (Shillingford et al., 2006).

In human embryonic stem cell-derived cardiomyocytes, PC2 was shown to regulate autophagy in a RyR2-dependent manner (Lu et al., 2017). The authors showed that PC2 knockdown reduced autophagic flux after glucose deprivation. In contrast, PC2 overexpression was able to increase autophagic flux under the same conditions. Glucose deprivation was shown to increase the binding of PC2 with RyR2 which was necessary for maintaining increased RyR2-mediated Ca^2+^ release after glucose deprivation. Finally, it was shown that this PC2-RyR2-mediated Ca^2+^ signaling induced autophagy by increasing AMPK activity, thereby inhibiting mTOR signaling.

## The RyR in Neurodegenerative Diseases

Impaired or altered autophagic flux is a hallmark of several neurodegenerative diseases but also of aging. This is a novel topic in the field of autophagy and has recently been extensively reviewed (Kroemer, 2015; Fîlfan et al., 2017; Gao et al., 2017; Karabiyik et al., 2017; Liu et al., 2017; Moloudizargari et al., 2017; Guo et al., 2018; Wang et al., 2018). Dysregulated RyR expression/activity also play important roles in the development of a number of neurodegenerative diseases. These emerging roles for the RyR in several neurodegenerative diseases will be highlighted in this part of the review article.

### Alzheimer’s Disease (AD)

In the hippocampus of several AD mouse models, RyR3 levels were shown to be upregulated (Chan et al., 2000; Chakroborty et al., 2012; Oules et al., 2012). This results in excessive RyR-mediated Ca^2+^ release, which underlies early pathological events in AD. Inhibition of RyRs using dantrolene reduced β- and γ-secretase activity and the phosphorylation of the amyloid precursor protein thereby lowering amyloid beta plaque formation (Chakroborty et al., 2012; Oules et al., 2012). It must be noted that also negative effects of dantrolene-mediated RyR inhibition in AD have been reported (Chami and Checler, 2014; Liu et al., 2014). This discrepancy was explained by the time point at which these RyRs were inhibited. Using a RyR3 knockout mouse model in a triple transgenic AD mouse model background, it was shown that RyR3 activity may have a protective effect during the early stages of the disease. Inhibiting RyR3 at this point would thus promote disease progression. In contrast, in older mice, RyR3 appears to promote disease progression, suggesting that blocking the channel at this time may decelerate the development of AD. Recently, it was shown that post translational modifications of RyR2 occur in SH-SY5Y cells overexpressing an AD linked amyloid precursor protein mutation (Lacampagne et al., 2017). These modifications lead to leaky RyR2 channels thereby elevating basal cytosolic Ca^2+^ levels. Stabilizing this RyR2-mediated Ca^2+^ leak resulted in a decrease in the processing of the amyloid precursor protein to amyloid beta. Similar results were reported using AD mouse models in which stabilizing leaky RyR2 channels by enhancing the binding of FKBP12.6 to RyR2 reduced amyloid beta plaque formation and improved synaptic plasticity (Lacampagne et al., 2017).

### Huntington’s Disease (HD)

RyR activity was also shown to be important in the development of Huntington’s disease (HD; Chen et al., 2011). Inhibition of RyRs by dantrolene in neurons obtained from a HD mouse model was shown to reduce glutamate-induced excitotoxicity. In addition, long term dantrolene feeding reduced the formation of pathological huntingtin aggregates and neuronal loss. Whether autophagy is also induced in these models after dantrolene treatment was not studied. It has been shown that compounds promoting autophagy by reducing IP_3_R-mediated Ca^2+^ release, thereby inhibiting calpain activation, are beneficial for treating HD (Williams et al., 2008). As calpain activity is critically regulated by intracellular Ca^2+^, inhibition of RyRs could also reduce calpain activity and in turn activate autophagy. However, further research will be needed to elucidate whether or not RyRs are involved in calpain-mediated autophagy regulation.

### Parkinson’s Disease

Parkinson’s disease is characterized by a loss of dopaminergic neurons, depletion of dopamine and mitochondrial dysfunction resulting in oxidative and excitotoxic stress (Hirsch et al., 2013). Recently, a new compound which showed neuroprotective effects in dopaminergic neurons, was tested in a Parkinson’s disease mouse model (Le Douaron et al., 2016). The neuroprotective effects of this compound could be partially counteracted by dantrolene, suggesting this compound operates in part by activating RyR-mediated Ca^2+^ signaling. This is in agreement with previous studies where caffeine and paraxanthine, a metabolite of caffeine, were shown to promote dopaminergic neuron survival and increased dopamine secretion (Guerreiro et al., 2008; Xu et al., 2010). The neuro-protective effect of paraxanthine was attributed to a mild activation of the RyR. It may be important that RyRs were only mildly activated in order to not sensitize these neurons to excitotoxic cell death, as was illustrated in a *Drosophila* model of Parkinson’s disease (Cassar et al., 2015). In this model, paraquat was used to induce oxidative stress, mimicking the disease. Reducing the amount of functional RyR channels in this model inhibited paraquat-induced cell death. Oxidative stress is a potent RyR sensitizer (Xu et al., 1998), which in turn could increase the sensitivity to excitotoxic stimuli. Reducing the amount of functional RyR channels may protect these cells from excitotoxic cell death.

### Spinocerebellar Ataxia

Spinocerebellar ataxia (SCA) is a progressive neurodegenerative disease of which several types are known to exist (Sun et al., 2016). Activation of autophagy was shown to be beneficial in the treatment of certain forms of SCA (Nascimento-Ferreira et al., 2011). SCA type 2 and 3 are characterized by loss of Purkinje cells due to aggregation of neurotoxic disease-causing mutant proteins. RyR activity is involved in the maturation of Purkinje cells (Ohashi et al., 2014). Increasing RyRs or RyR activity could potentially rescue the loss of Purkinje cells. This was explored in a SCA3 mouse model where increasing serotonin and RyR levels in the cerebellum rescued the loss of Purkinje cells associated with the disease (Hsieh et al., 2017). In a mouse model for SCA2 the mutant ataxin-2 protein was shown to trigger IP_3_R-mediated Ca^2+^ release, which was amplified by RyR activity via CICR (Liu et al., 2009). Treatment of these mice with dantrolene protected Purkinje cells from excitotoxicity and cell death. These studies indicate that the RyR may be a potential target for treating certain types of SCA.

## Conclusion

**Figure 1:**
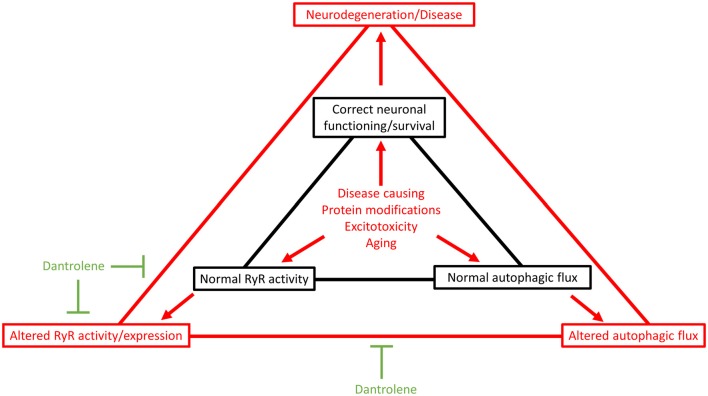
The interplay between ryanodine receptors (RyRs) and autophagy contributes to neuronal function and health. Recent studies linked RyR signaling to autophagy suggest that RyR function and autophagy co-operate to maintain neuronal health (indicated in black). Disease causing mutant proteins, excitotoxicity and aging are known causes of neurodegenerative diseases. It is becoming increasingly clear that during the onset of these diseases changes in RyR signaling and autophagy occur contributing to the disease. These changes in RyR signaling may also affect the regulation of autophagy thereby accelerating even further the disease progression (indicated in red). Because of the link between RyR-mediated Ca^2+^ signaling and autophagy, the RyR inhibitor dantrolene could potentially via inhibiting RyRs also regulate autophagic flux. In this way, dantrolene may have multiple therapeutic effects (indicated in green). This may in part explain the beneficial effects of dantrolene treatment for several neurodegenerative diseases.

RyR signaling and autophagy are two factors critically linked to the correct functioning and survival of neurons (Figure 1, indicated in black). Studies showing how RyR-signaling regulates autophagy have only recently emerged making this a novel avenue in the autophagy field. In addition, several RyR-interacting/-modulating proteins have known roles in the regulation of autophagy which have not (yet) been linked to regulating RyR-mediated Ca^2+^ release. It will be interesting to see whether these interactors also influence autophagy by modulating RyR-mediated Ca^2+^ release.

Alterations in RyR-mediated Ca^2+^ release and autophagy have also been shown to contribute to several neurodegenerative diseases. Disease causing mutant proteins, excitotoxicity and aging are not only the cause for these diseases but also change RyR function and autophagy. As RyR function and autophagy are involved in maintaining neuronal health, changes in these processes may result in aggravating disease progression (Figure 1, indicated in red). Inhibition of RyR-mediated Ca^2+^ release has been shown to confer beneficial effects in treating a number of these diseases. Several of the above mentioned studies used the FDA-approved drug dantrolene in order to block RyR-mediated Ca^2+^ release. An observation common in the studies looking at how RyRs regulate autophagy, is that RyR inhibition via dantrolene changes/normalizes autophagic flux. This may in part underlie the beneficial effects of dantrolene in several neurodegenerative diseases as by inhibiting RyR activity it may also regulate autophagic flux indirectly (Figure 1, indicated in green). However, more research will be needed to gain further insights on the role of RyRs in regulating autophagy in the context of neurodegenerative diseases.

## Author Contributions

TV wrote this review article.

## Conflict of Interest Statement

The author declares that the research was conducted in the absence of any commercial or financial relationships that could be construed as a potential conflict of interest.
